# Pertussis Reinfection in an Adult: A Cause of Persistent Cough Not to Be Ignored

**DOI:** 10.1155/2017/4786141

**Published:** 2017-07-13

**Authors:** Theocharis Koufakis, Anastasia Paschala, Dimitrios Siapardanis

**Affiliations:** ^1^Department of Internal Medicine, General Hospital of Larissa, 1 Tsakalof Street, 41221 Larissa, Greece; ^2^George Papanikolaou General Hospital, Exochi, 57010 Thessaloniki, Greece; ^3^Department of Internal Medicine, St. Josef Hospital, Robert-Koch-Straße 16, 42781 Haan, Germany

## Abstract

Pertussis is traditionally considered as a disease of the childhood; however, accumulating evidence suggests a stable increase of its incidence among adults and adolescents, during the last decades. Despite the fact that reinfection after natural disease or vaccination is not uncommon, the index of clinical suspicion of pertussis diagnosis in adults remains low. In this article, we report a case of pertussis reinfection 30 years after natural infection, which was complicated by pneumonia, and we discuss our diagnostic and therapeutic approach, aiming to raise clinicians' degree of suspicion regarding pertussis diagnosis in adults. Prompt recognition and appropriate therapy of adult patients can result in the effective control of the symptoms, prevention of severe complications, and spread of the infection to children; thus, they are of great clinical and public health importance.

## 1. Introduction

Pertussis (also known as whooping cough) is a respiratory tract infection, caused mainly by the gram-negative, aerobic, pathogenic, encapsulated coccobacillus* Bordetella pertussis*. Still, a similar to pertussis clinical entity caused by* Bordetella parapertussis* has been also described in humans [1]. The disease is considered to be highly contagious. Its main clinical manifestation is the paroxysmal cough, which, in some cases, can be extremely torturous for the patient, since it can last for weeks and be accompanied by apnea, exhaustion, and posttussive vomiting [2].

Despite the fact that most countries have adopted extended vaccination programs against the disease, pertussis continues to be a significant public health challenge. Important differences in mortality rates among very young, unimmunized infants, depending on whether or not adequate supportive care is available, have been observed [3]. Although it is traditionally considered as a disease of the childhood, accumulating evidence suggests a stable increase of pertussis incidence among adults and adolescents, during the last decades [4].

Reinfection after natural disease or vaccination is not uncommon [5]; however, the index of clinical suspicion of pertussis diagnosis in adults remains low. While the duration of protection in immunized subjects seems to be shorter for those immunized with acellular pertussis vaccines, protection offered by whole-cell pertussis vaccines is not lifelong [5]. Immunological protection against the typical disease starts to wane 3 to 5 years and nearly disappears approximately 12 years after vaccination [6]. In this article, we report a case of pertussis reinfection 30 years after natural infection, which was complicated by pneumonia, and we discuss our diagnostic and therapeutic approach, aiming to raise clinicians' degree of suspicion regarding pertussis diagnosis in adults.

## 2. Case Presentation

A 40-year-old female patient presented with complaints of prolonged, paroxysmal cough for the last 4 weeks. The cough was followed by gasping for breath and rarely vomiting. She was obese (body mass index 41 kg/m^2^) and had a history of recently diagnosed Diabetes Mellitus Type 2, for which she was on metformin, 500 mg, orally, twice a day. She had received a 7-day course of amoxicillin-clavulanic acid (1000 mg, twice a day) and inhaled fluticasone, with no clinical response.

**Figure 1:**
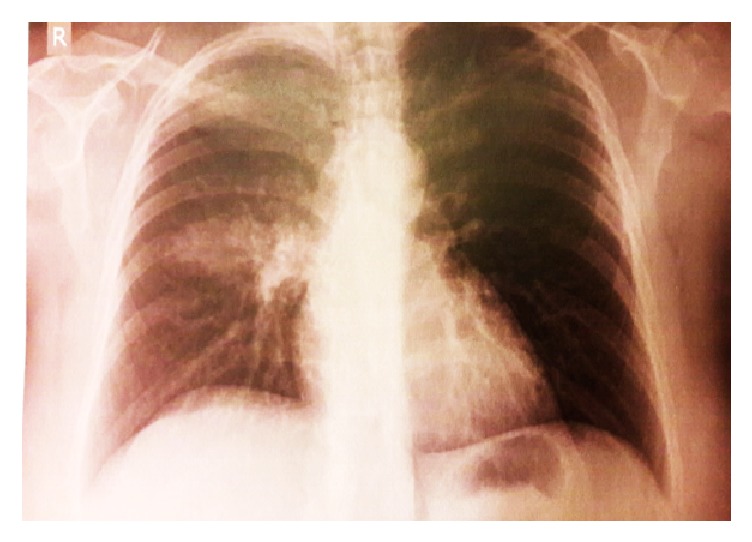
Patient's chest X-ray demonstrating a right upper lobe consolidation.

On physical examination, she was looking fatigued and had low grade fever (37.4°C) and mild sinus tachycardia (95 beats per minute), while her blood pressure and oxygen saturation were normal (140/90 mmHg and 98%, resp.). On lung auscultation, chest sounded clear. Her initial laboratory evaluation was normal, apart from elevated C-Reactive Protein (CRP) (Table 1). However, her chest X-ray demonstrated a right upper lobe consolidation (Figure 1).

After looking back at her personal medical record, we discovered that the patient had undergone a pertussis infection, at the age of ten. The original diagnosis had been confirmed by a positive result for* Bordetella pertussis* nasopharyngeal aspirate specimen culture and the disease had been treated with erythromycin. She had been never vaccinated against the disease, as confirmed by her medical record documentation and the patient's mother who was also interviewed.

Further laboratory work-up revealed negative urine antigen tests for* Streptococcus pneumoniae* and* Legionella pneumophila*, negative immune serologic tests for* Mycoplasma pneumoniae*,* Chlamydophila pneumoniae*, and* Coxiella burnetii*, and negative urine and blood cultures. Nasopharyngeal samples for culture and PCR for pertussis were not taken early in the course of infection, due to lack of suspicion of pertussis. The diagnosis of acute pertussis infection was established by high titer (120 ELISA units/ml) of IgG antibodies against Pertussis Toxin (PT). The serological testing was performed in a private laboratory, with a commercial assay for measuring IgG anti-PT antibodies (Savyon Diagnostics, Ashdod, Israel), accredited according to European (EN) 15189 standard, as proposed by the European Center for Disease Prevention and Control (ECDC) guidelines for pertussis laboratory diagnosis [7]. The patient was treated with a 7-day clarithromycin course (500 mg, twice a day), which resulted in the control of her symptoms within the next 2 weeks. No other similar case was reported among the patients' relatives or coworkers; therefore, the source of transmission remains unknown. One month after recovery, she started a full vaccination course with tetanus-diphtheria-pertussis (Tdap) combination vaccine. In her follow-up visits she remained free of symptoms and in good physical condition, while her chest X-ray returned to normal.

## 3. Discussion

The classical description of the clinical course of pertussis includes three stages: catarrhal, paroxysmal, and convalescent. These stages are met in the majority of the cases. Still, in older or immunized patients, some attenuation of symptoms (especially of paroxysmal stage) can be observed [8]. In adults, the clinical picture may be atypical and the predominant symptom is the prolonged cough, which, in 70 to 90% of the cases, is paroxysmal in nature [4]. This atypical clinical course often results in the underdiagnosis or misdiagnosis of the disease in adults, as postviral cough, asthma, or chronic sinusitis [9, 10]. Delay in the diagnosis can be proved hazardous, given that almost 1 in 4 adults will present complications, such as pneumonia (10%), pneumothorax, rib fracture, hernia, seizures, and syncope, among others [11].

The gold standard method for the establishment of the diagnosis is the isolation of the pathogen from cultured tissues or fluids, mainly nasopharyngeal swabs, aspirates, or washes. Yet, several confounding factors can influence the reliability of the method, such as treatment with antibiotics, the stage of the disease, and previous vaccination history [12]. Detection of bacterial nucleic acids through Polymerase Chain Reaction (PCR) is an alternative method, which is preferable in school-age children, adolescents, and adults, with a sensitivity varying between 10 and 30% [13]. Serology may also be useful, particularly in cases where nasopharyngeal samples for culture and PCR are not taken early in the course of infection, due to lack of suspicion of pertussis. A 4-fold increase in anti-PT IgG with 4–6-week intervals is probably the most reliable serologic test [6]. Alternatively, a single measurement reaching an anti-PT IgG level of >94–110 EU/mL has been suggested as the diagnostic cutoff point for recent infection [14]. Several commercial assays for measuring IgG anti-PT are available in the European Union [7], and some of these have been tested and found to be appropriate as compared to an in-house ELISA [15]. On the other hand, there is no commercial kit approved by the United States Food and Drug Administration (FDA) for pertussis diagnosis. Notably, as happened in the presented case, leukocytosis is generally uncommon in adolescents, adults, and partially immunized children and even in the absence of it, the diagnosis of pertussis should not be excluded [16].

Antibiotic agents of choice for pertussis treatment are macrolides, such as erythromycin, clarithromycin, and azithromycin. Appropriate antibiotic treatment can eliminate* Bordetella pertussis* from the respiratory tract and, consequently, prevent transmission to susceptible contacts. Furthermore, it has been proved that antibiotics decrease the probability of secondary bacterial infections and reduce duration and severity of symptoms, when given early in the course of the disease [17].

Of major interest, our patient's natural infection in childhood had been treated with erythromycin. Unfortunately, we were not able to document the exact timing of antibiotic treatment. Still, it has been suggested in the literature that macrolides administration can weaken the subsequent immune response to infections, if given early during the course of the disease [18]. Therefore, this macrolides effect may have potentially contributed to our patient's susceptibility to pertussis reinfection.

Newborns are vulnerable to infection during the first weeks of their life, given that the quantity of maternal antibodies transferred is, in most cases, insufficient to provide protection [11]. Relevant studies suggest that adult household members, including parents and grandparents, can be identified as the source of the microorganism transmission to children in approximately 22% of the cases [19]. Therefore, early identification of diseased adults is of great value, since it can lead to the effective termination of pertussis' chain of infection. Tdap boost vaccination for subjects older than 11 years is an effective prevention strategy and, therefore, should not be omitted.

It has been estimated that nearly 10 to 20% of adults with cough lasting more than a week, suffer from pertussis [20]. Similar symptoms can be caused by other pathogens, as well, including adenoviruses, respiratory syncytial viruses (RSV), human parainfluenza viruses, influenza viruses,* Mycoplasma pneumonia*, and rhinoviruses [9]. Coinfections, particularly with* Bordetella pertussis *and RSV, are commonly seen among infants [13]. In adult patients, it is essential that the differential diagnosis of persistent cough should include primary and secondary pulmonary malignancies, and imaging with X-ray or Computed Tomography (CT) of the chest must be accordingly performed in these cases [21].

## 4. Conclusion

This report aims to highlight the fact that pertussis is not only a disease of the childhood, but it should be also suspected in adults, presenting with chronic cough, even if they do have a previous history of natural infection or vaccination. Prompt recognition and appropriate therapy of adult patients can result in the effective control of the symptoms, prevention of severe complications, and spread of the infection to children; thus, they are of great clinical and public health importance.

## Figures and Tables

**Table 1:** Patient's main laboratory findings on presentation. Notably, leukocytosis is generally uncommon in adults and even in the absence of it, the diagnosis of pertussis should not be excluded.

Parameter (units, reference range)	Value
White blood cell (10^3^/*μ*L, 4–11)	10.2
CRP (mg/dl, <0.8)	3.2
Alanine aminotransferase (IU/L, <40)	24
Lactate dehydrogenase (IU/L, <350)	141
Creatine phosphokinase (IU/L, <100)	74
